# Localisation of Unmanned Underwater Vehicles (UUVs) in Complex and Confined Environments: A Review

**DOI:** 10.3390/s20216203

**Published:** 2020-10-30

**Authors:** Simon Watson, Daniel A. Duecker, Keir Groves

**Affiliations:** 1 Department of Electrical and Electronic Engineering, The University of Manchester, Manchester M13 9PL, UK; keir.groves@manchester.ac.uk; 2 Institute of Mechanics and Ocean Engineering, Hamburg University of Technology, 21073 Hamburg, Germany; daniel.duecker@tuhh.de

**Keywords:** unmanned underwater vehicle (UUV), localisation, complex environments

## Abstract

The inspection of aquatic environments is a challenging activity, which is made more difficult if the environment is complex or confined, such as those that are found in nuclear storage facilities and accident sites, marinas and boatyards, liquid storage tanks, or flooded tunnels and sewers. Human inspections of these environments are often dangerous or infeasible, so remote inspection using unmanned underwater vehicles (UUVs) is used. Due to access restrictions and environmental limitations, such as low illumination levels, turbidity, and a lack of salient features, traditional localisation systems that have been developed for use in large bodies of water cannot be used. This means that UUV capabilities are severely restricted to manually controlled low-quality visual inspections, generating non-geospatially located data. The localisation of UUVs in these environments would enable the autonomous behaviour and the development of accurate maps. This article presents a review of the state-of-the-art in localisation technologies for these environments and identifies areas of future research to overcome the challenges posed.

## 1. Introduction

The use of unmanned underwater vehicles (UUVs) has revolutionised aquatic inspection over the last 30–40 years. UUVs are aquatic robots and are covered by two broad categories, Remotely Operated Vehicles (ROVs) or Autonomous Underwater Vehicles (AUVs). ROVs are remotely operated by a human and often tethered. AUVs have varying levels of autonomous capabilities and they do not require either human operation or often a tether [1].

In the open ocean, UUVs are used widely for the inspection and maintenance of subsea assets, surveys, oceanography, and military purposes [2,3,4,5]. They are also now being extensively used for civil and onshore inspections, such as nuclear storage facilities [6], ship hull inspections [7], the inspection of flooded sewers and mines [8], and liquid storage tank inspections [9,10].

A critical component of any robot is the localisation system. At the simplest level, a manual operator can use a camera to navigate around an environment. This approach does not automatically generate a map and it relies on the operator being able to identify salient features to localize themselves. Areas of interest may be marked on an independent map by the operator.

Enabling accurate geo-spatial localisation of sensor data requires the robot to have its own on-board localisation system. This is also a pre-requisite for autonomous behavior. Localisation technologies and algorithms have been a topic of rich research development over the last decade for ocean-going UUVs and there are several excellent reviews that discuss their capabilities and limitations [11,12,13]. However, there are significant additional challenges when trying to localize in more complex and confined environments. These include, but are not limited to, a lack of external infrastructure, low illumination levels, high turbidity, and a lack of salient features.

This article presents a number of contributions. Firstly, what constitutes a complex and confined underwater environment will be defined and a number of specific application use cases will be introduced. UUVs that have been specifically developed to explore these environments will then be presented. Existing localisation technologies will then be presented and reviewed from the perspective of their suitability for use in complex and confined environments. Finally, conclusions will be drawn regarding the existing technological landscape and where further research may be needed.

This article is structured, as follows: Section 2 will formulate the problem by identifying and analysing a range of complex and confined environments. This section will also discuss the operational requirements for inspections and introduce UUVs that have been developed for use in them. Section 3 will then present the review of the main localisation technologies, whilst Section 4 will provide a comprehensive analysis and discussion of the technologies in the context of the environments from Section 2. Section 5 presents conclusions and areas of potential future research.

## 2. Problem Statement

The problem statement on the localisation of UUVs in complex environments can be formulated in three stages: the identification of the target environments; identification of mission scenarios that are required to be undertaken; and finally, the identification of suitable underwater vehicles that could be used to complete the missions.

### 2.1. Terminology and Reference Frames

**Figure 1:**
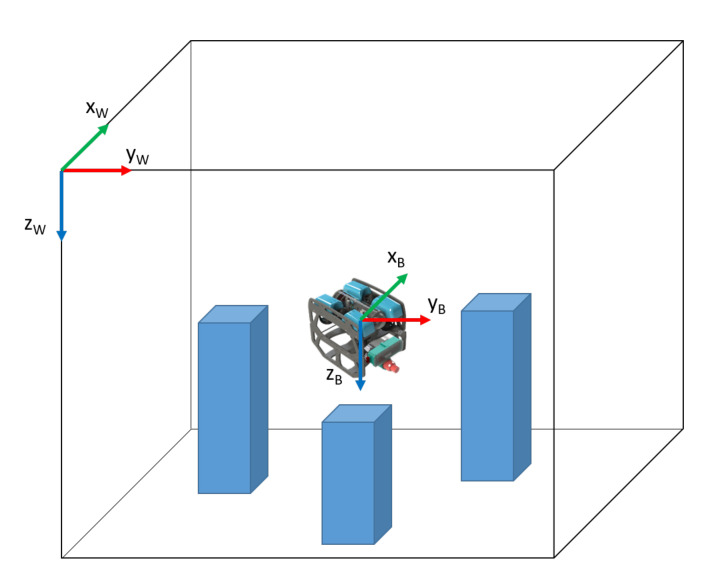
World-fixed and Body-fixed Axis Frames.

There are many different terms used to describe aspects of localisation systems. Within this article, we will define and use the following:

- **Absolute Localisation**—in a complex environment, true global positioning (a position estimate relative to the earth centred, earth-fixed reference frame for example) is often not possible and not usually relevant. Due to the high ferro-magnetic content of structures in complex environments, magnetic navigation is also often not feasible. The most appropriate alternative is to reference to a world-fixed reference frame, which is likely to be defined by the boundaries of the environment or the position of external beacons, markers or features of the fixed environment, whose position is known, as shown in Figure 1. This type of localisation is known as Absolute Localisation.
- **Relative Localisation**—this form of localisation estimates changes in the robot’s body frame relative to an arbitrary point in the environment. Relative localisation methods primarily generate velocities rather than position fixes. Dead reckoning may then be used the calculate the location relative to a position fix. If exteroceptive sensors are used, such as cameras, localisation could be relative to several features in the environment.

Unfortunately, some systems do not easily fall into these categories. For example, simultaneous localisation and Mapping (SLAM) systems provide a position estimate relative to a map that evolves throughout the mission. Once the complete map is built and is essentially static, SLAM can be considered as an absolute position estimate.

For the analysis in this article, we will consider any system which can give a position fix with respect to external beacons, infrastructure, or environmental features as providing an absolute position estimate. Any system that primarily delivers a velocity or change in position in either the robot’s body frame or a worldfixed frame is considered to be a relative position estimate.

### 2.2. Application Areas and Environmental Features

There are a number of application areas that have complex environments that require inspection or operations within. These environments will have characteristics that will have direct impact on the usability of different localisation technologies.

#### 2.2.1. Application Areas

Nine application areas have been identified for consideration in this article.

(A) **Modern Nuclear Storage Ponds**—there are over 1000 wet nuclear storage facilities globally which require continual monitoring. These are static, structured environments with clear water and good illumination levels to facilitate visual inspections [14]. These facilities are usually indoors and can be a few meters in dimension, up to 50 m × 25 m × 10 m.
(B) **Legacy Nuclear Storage Ponds**—legacy nuclear storage ponds are those that were constructed in the 1950’s and 60’s and which have operated well beyond their original lifespan. There are only a few of these globally, however they present significant decommissioning challenges [15]. Many of these facilities are outdoors and open to the elements and can be up to 50 m × 25 m × 10 m in size.
(C) **Legacy Nuclear Storage Silos**—as well as large ponds, nuclear waste was also stored in silos in the 19050’s and 60’s, such as those found on the Sellafield site in the UK [16]. These are around 5 m in diameter and 16 m deep and contain nuclear material stored in water.
(D) **Nuclear Reactor Pressure Vessels**—reactor Pressure Vessels (RPVs) are a critical component of nuclear reactors and they require periodic inspection to ensure there are no structural defects [17,18]. In 2011, the incident at the Fukushima Daiichi Nuclear Power Plant, Japan, led to the fuel rods melting through the RPV into the flooded pedestal below. This led to the formation of a highly complex and radioactive aquatic environment with very restricted access, which requires investigation [19].
(E) **Offshore Asset Decommissioning**—there are a significant number of offshore assets globally, primarily associated with energy generation; either Oil & Gas (O&G) or wind. Over the next 30 years, many of these will need to be decommissioned [20,21]. The decommissioning process is likely to generate a range of complex environments which UUVs will have to operate in.
(F) **Ship Hulls**—the inspection of ship hulls is very important in the maritime industry as structural defects can lead to significant reductions in revenue. Traditionally, inspections have been conducted in dry-docks or by divers, which is both expensive and dangerous [22]. UUVs have recently been developed to undertake inspections of both active ships [7] and ship wrecks [23].
(G) **Liquid Storage Tanks**—liquid storage tanks are widely used all over the world and periodic inspection is required to ensure that structural defects are not present which could lead to catastrophic failures [9]. If the tanks are used to store water, inspections are also required to ensure the water quality is kept high [24,25]. These inspections can be undertaken when the tank is empty (dry) or full (wet). Wet inspections are lower cost and they can be conducted by UUVs; however, they are often limited to manual inspections/interactions due to a lack of localisation technologies. UUVs have also been used to inspect water ballast tanks on ships [26,27].
(H) **Marinas, Harbours and Boatyards**—Marinas, harbours, and boatyards are vital areas that allow for the use of yachts and small boats. Periodic inspections are required to ensure there are no structural defects and, more recently, for security purposes [28]. Often, these inspections are conducted by divers, which is very hazardous [29]. UUVs have started to be developed to replace divers for these inspections [30].
(I) **Tunnels, Sewers, and Flooded Mines**—there are 1000 s of km of flooded mine shafts, tunnels, waterways and sewers globally which require inspection to ensure their continued safe operation [8,31,32]. Many of these areas have never been inspected and UUVs offer a safe option to do this. The environments may be natural formations which have been re-purposed, or man-made constructions.

#### 2.2.2. Environmental Characteristics

Eleven environmental characteristics have been identified for consideration in this article. The application areas that are described in Section 2.2.1 will contain subsets of these.

(i) **Scale**—these are the characteristic dimensions of the environment which needs to be explored. This size of the environment will have a direct impact on the size of the UUV that can be deployed and the accuracy the localisation system has to provide. Dimensions are given in meters as either [width × length × depth] or [ϕ (diameter) × depth].
(ii) **Obstacles**—the majority of the applications can be considered as vessels or facilities which contain water. They will be bounded by a floor, walls, and often a ceiling. Obstacles are defined as objects that are not part of this bounding infrastructure. If they are free-standing, they will likely be placed on the floor. Alternatively, they may be a significant protrusion from one of the surfaces.
(iii) **Structure**—if there are obstacles in the environment, they can be defined as either structured or unstructured. Structured means that they have been placed in the environment in an ordered manner, such as containers that have been stacked. Unstructured means that there is no order to the placement of them. An environment can have unstructured obstacles, even if they have been purposely plac ed and there is clear knowledge of where they are.
(iv) **Obstacle Type**—if there are obstacles, they can also be classified as static or dynamic. Static obstacles are fixed and they will not move for the duration of a mission. Dynamic obstacles will move during the mission.
(v) **Access**—two methods of access will be considered: surface deployment and restricted access. Surface deployment is where there is no ceiling to the environment and the UUV can be deployed directly into the water from the edge. Restricted access is where there is a ceiling and the UUV needs to be deployed through a hatch or similar entry port.
(vi) **Additional Infrastructure**—some environments will allow for additional infrastructure to be placed in them. Quite often, these are the more open environments, where, for example, beacons could be installed around the edges. Other, more closed environments with restricted access, do not allow this.
(vii) **Line-of-sight (LoS)**—if an environment has obstacles, their disposition may inhibit LoS from the UUV to various points. For this analysis LoS will be considered to the surface and to the edges of the environment.
(viii) **Turbidity**—urbidity is a measure of water clarity and is affected by the presence of suspended particulates. Light is scattered by the particles, so the more there are, the more light is scattered and the higher the turbidity. If there are no particles, then the turbidity is very low and the water will be clear.
(ix) **Ambient Illumination Levels**—some environments will have external light sources that will provide a certain level of ambient lighting (i.e., not provided by lights mounted on the UUV). Other environments will not and the only light will be generated from on board the UUV.
(x) **Salient Features**—certain localisation technologies require the identification of features in the environment. Detectable features are known as salient features. A long smooth, uniform surface will provide no salient features; however, if you placed defined objects, such as QR codes, on the wall, then these could then be detected.
(xi) **Variance of Environment**—whilst objects have been defined as static or dynamic for the duration of a single mission, the variance of the environment over a number of missions also needs to be considered. Some environments will not change over an extended duration (years), whereas others will change over hours or days.

#### 2.2.3. Analysis

Table 1 shows an analysis of different application environments. Each environment has a different sub-set of the environmental characteristics, which suggests that no single localisation system is likely to be applicable to all environments. The scales of the environments mean that the accuracy of the localisation system will have to be much smaller than those that are commonly found in ocean going systems.

### 2.3. Missions

UUV missions in the environments that are identified in Section 2.2 can broadly be described as inspection, maintenance and repair (IMR). Because of technological limitations, the majority of the missions are inspections [33], however there are an increasing number of maintenance and repair activities being undertaken. Operations are normally manual, however more autonomous capabilities are being developed [34].

UUV operation is not a binary choice between fully manual or fully autonomous, rather there are different levels of autonomy (LoA) [35]. For this work, we will adopt and adapt the six levels of autonomy proposed for surgical robots in [36].

0. **No Autonomy**—the UUV is entirely tele-operated by a human.
1. **Robot Assistance**—the UUV provides some automated functionality, for example staying at a set depth (set by the operator) or prohibiting the operator to maneuver into obstacles. The operator is still in full control of the UUV.
2. **Task Autonomy**—the UUV is able to execute motions under the guidance of the operator. For example way-points could be set to which the UUV will travel with no further input from the operator.
3. **Conditional Autonomy**—the UUV generates task strategies, but requires a human to select which one to undertake. For example, when exploring an environment, the UUV may identify several different routes to take, with the human selecting the most appropriate one.
4. **High Autonomy**—the UUV can plan and execute missions based on a set of boundary conditions specified by the operator. The operator does not require to select which one the UUV should do, however they are there to oversee the task execution.
5. **Full Autonomy**—the UUV requires no human input at all. It is deployed into the environment and left with no operator oversight.

Table 2 shows current mission activities that are undertaken by UUVs and their levels of autonomy. Inspection tasks are currently conducted in all environments except for modern nuclear storage facilities. Limited maintenance and repair activities are conducted in legacy nuclear storage ponds, liquid storage tanks and on offshore assets. The highest level of autonomy achieved is 1, where the UUV is able to automate basic functions, such as staying at a given depth. There are no autonomous activities undertaken in any of the environments.

Table 3 shows the predicted activities and levels of autonomy in the future. The highest level of autonomy, 5, is the aspiration for robotics in general; however, there are still significant technological and regulatory challenges to overcome.

There are a number of environments that may be suited to allow the highest level of autonomy. Modern nuclear storage ponds may seem an unusual application for allowing high levels of autonomy, given their stringent safety requirements, however they are highly structured and static environments with low turbidity, good ambient lighting, and clear lines of sight to the surface. This means that UUVs could operate more easily within them, as long as the appropriate verification and validation of their behaviour has been conducted.

The inspection of ship hulls and liquid storage tanks may also aspire to the highest level of autonomy as the environments are usually static with few external obstacles. For sewers and flooded mines, the size (length) and communications challenges may dictate that UUVs have to have a much higher level of autonomy, as operator control/intervention may not be possible. 

Due to the nature of the material in legacy nuclear storage ponds (radioactive sludge, material of unknown composition), a lower level of autonomy will be required, where there is operator oversight (level 4). 

Offshore asset decommissioning and marinas, harbours and boatyards are all highly complex environments with a significant numbers of obstacles. As the environments may be dynamic, a lower level of autonomy may be utilised, where operators select appropriate routes and missions. Legacy nuclear storage silos are likely to continue to be manually inspected due to the very high level of obstacle complexity in them.

#### Analysis

To increase the level of autonomy of a UUV requires an increase in its sensing and perception capabilities. The key piece of information that is required is the UUVs position within its environment, which is necessary to plan missions or navigate safely around the environment. As Table 2 shows, all of the existing applications have UUVs with levels of autonomy 0 or 1. Automated depth position control is often used to assist operators who can then focus on controlling movement in the x-y plane [37].

Inspection tasks will likely only require an absolute position estimate, however maintenance and repair activities will require both absolute and relative. Absolute position estimates would allow for higher levels of autonomy, however it would also be very useful when operating at levels 0 or 1. Positional feedback to an operator would improve inspection performance as well as providing accurate positions of inspection data.

The absolute and relative position estimates are likely to have different accuracy requirements, which will be discussed in more detail in Section 3.

### 2.4. UUVs

**Figure 2:**
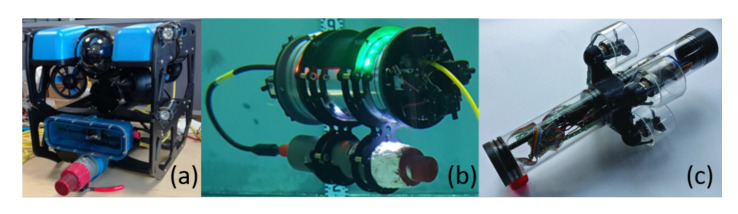
Unmanned underwater vehicles (UUVs): (**a**) BlueROV2 [38], (**b**) AVEXIS [19], and (**c**) HippoCampus [39].

The UUVs identified for this article can be categorised as either commercial or research. All current inspection operations are manual, therefore the commercially available UUVs are ROVs, as identified in Section 2.3. Some of the research platforms identified are being developed for increased levels of autonomy and therefore have both ROV and AUV abilities. Figure 2 shows a number of commercial and research UUVs and Table 4 shows key characteristics of a range of platforms.

Table 4 shows that the commercial UUVs all have a small form factor, with the largest being the BlueROV2, which is 0.46 m long. This means that the they could be deployed into restricted access areas with small entry ports; however, the size limits the payload that can be carried. All of the commercial platforms come with on-board cameras and lights and they can be fitted with a small manipulator, sonar, or other perception systems; however, the total additional payload appears to be limited to 1 kg. The BlueROV2 and the DTG3 both have tetherless capability (AUV), whereas the VideoRay and AC-ROV are only tethered.

For the research platforms, UX-1 is the largest and it is able to carry a range of scientific equipment, such as radiation sensors, multi-spectral cameras, and water samplers [40]. The tether is used for communications rather than power (although power can also be sent down it) and the vehicle can operate for up to five hours. Both the AVEXIS and HippoCampus UUVs have very small form factors and limited payloads and they have been developed for visual inspections of nuclear facilities [19] and marinas [39], respectively.

#### Analysis

The range of UUVs that are suitable for missions in confined and hazardous environments is relatively limited. A key characteristic of them is their small form factor when compared to ocean-going UUVs. This means that there is reduced capacity to carry additional payload. Many ocean-based localisation systems are quite large and they could not be used by these platforms.

## 3. Localisation Technologies

Underwater robot localisation technologies are primarily based on one of four fundamental physical principles: acoustic, electromagnetic, inertial, and vision. In this section, we will review existing localisation technologies derived from these physical principles. For the purposes of the initial review and analysis, the technologies are considered in isolation; however, as discussed in Section 4, in practice two or more methods would often be used in unison. A comprehensive summary is depicted in Table 5 and Table 6 in Section 4.

### 3.1. Inertial Navigation

Inertial navigation is the process of determining the position and velocity of a vehicle from measurements of angular velocity and linear acceleration, according to the laws of classical mechanics [41]. An inertial measurement unit (IMU) is used to measure linear acceleration and angular velocity, which typically comprises three orthogonally mounted accelerometers and three orthogonally mounted gyroscopes [42]. Given the ability to measure the acceleration of a body, it is possible to calculate changes in velocity by integrating the acceleration with respect to time, and changes in position can be determined by a second time integration.

To be able to navigate in 3D space, it is necessary to keep track of the direction in which the accelerometers are pointing. The gyroscopes in an IMU measure rotational velocity and, by integrating their readings with respect to time, changes in direction can be logged. It is possible to determine velocities and displacements in the global frame by rotating acceleration measurements into a fixed global reference frame prior to integration. Inertial navigation systems track changes in position, so the position of an object can only be determined relative to a known starting pose and velocity; this is known as deadreckoning.

One of the key benefits of inertial navigation is the fact that the accuracy and precision of the position estimate are largely unaffected by external factors, such as light levels or acoustic reflections. Because the IMU is measuring in an inertial frame that is not linked to the environment, it is unaffected by disturbances, such as water currents. Unlike many other sensing methods discussed in this section, an IMU is not reliant on environmental features for localisation and the accuracy of the measurement has no relation to the size of the environment. Internal noise and bias in a high quality IMU is generally well specified and it can be taken into account in sensor fusion applications. Modern IMUs generally provide data with a fast refresh rate (≥100 Hz) and minimal latency.

The overriding source of error in inertial navigation applications comes from the propagation of orientation errors that are caused by noise and in-run bias of the gyroscope signals [43]. An error in orientation means that acceleration due to gravity cannot be correctly removed from the accelerometer signals and any error in removal of this relatively strong acceleration vector is integrated twice and causes exponential drift. Errors in orientation also cause the derived linear accelerations to be integrated in the wrong directions; however, in the case of aquatic vehicles, the linear accelerations are generally around an order of magnitude that is smaller than gravity (9.81 $m/s^{2}$). Titterton [42] finds that an unaided tactical grade IMU will accumulate position error at approximately 100 km/h and a navigation grade IMU will accumulate position error at approximately 1 km/h.

#### Suitable Hardware

There are several different technologies that are commonly used as the basis for IMUs, such as ring lasers, fibre optics, and microelectromechanical systems (MEMS). In recent years, the rapid development of MEMS technologies has meant that high accuracy IMUs are now much more affordable and they are available in much smaller packages [44]. MEMS based IMUs are generally better suited to use in the robots and applications presented in Section 2, due to their size, cost, and rapid advancements in accuracy. For instance, it is currently possible to purchase a six-axis tactical grade MEMS IMU that comes in a 5 cm × 5 cm × 1 cm package, costs approximately 3K GBP, and has a gyro in-run bias stability of 0.8 deg./hour [45].

Fibre optic IMUs represent the next step up in terms of accuracy, cost and size. The KVH1750 is acommonly used small form factor navigation grade fibre optic IMU. It is a cylindrical unit that is 7 cm tall, has a diameter of 9 cm, costs approximately 15K GBP, and has a gyro in-run bias stability of 0.05 deg./hour [46]. The size and cost of a fibre optic IMU is high when compared to the size and cost of the UUVs detailed in Section 2.4 and, therefore, would only be suitable in circumstances where it is not possible to use other aiding sensors.

Magnetometers are, in some cases, used in conjunction with the inertial sensors to reduce drift. However, they can be heavily affected by noise and external disturbance from ferromagnetic material in the environment, such as obstacles or infrastructure. Interference can also be generated by electrical components such as electric motors, so they are therefore not suited to small vehicles where the thrusters are necessarily close to sensing components.

### 3.2. Dynamic System Models

A 6 DOF mathematical dynamic model can be used in order to simulate the motion of an aquatic vehicle: velocities can be predicted from the control inputs. Therefore, the dynamic model can also be used to localise the vehicle relative to a known starting pose and velocity.

The dynamic model is usually represented by a set of equations of motion that are constructed according to Newtons second law. Fossen gives the standard format of the equations of motion for marine vehicles [47] and describes how the equations are assembled.

Given the control inputs and the initial conditions, the equations of motion can be numerically solved using a time stepping solver to give velocity predictions [48]. These body frame velocities can then be transformed into a global reference frame and integrated over a time period to give a change in position. As with inertial navigation, navigation using a dynamic model can only track changes in displacement and, therefore, it is defined as a deadreckoning method.

The output of the dynamic system model is free from noise, because it is a simulation. There are no environmental sensors used; therefore, there is no susceptibility to external signal interference, such as from acoustic or electromagnetic sources. However, there are several sources of error. The two main sources are external disturbances, such as those from water currents or tethers, and imperfections in the mathematical model [49]. A dynamic model alone can only localise relative to the water that surrounds the vehicle, so it cannot account for currents or any other movement of the water column [50]. Unmodelled hydrodynamic parameters or incorrectly identified coefficients cause the breakdown of the dynamic model during certain manoeuvres. Without an accurate model, localisation estimates will not be valid. Additionally, a dynamic model will produce incorrect predictions if there are changes to the vehicle or environment, such as an under-performing thruster or a change in fluid density [51].

Localising from a dynamic system model accumulates error in a similar way to an inertial navigation system. Therefore, in anything but ideal conditions, where there are no external disturbances, a dynamic model will lose accuracy in a short time period if unaided.

#### Suitable Hardware

The dynamic model requires no dedicated sensing hardware, as it uses the thruster command signals and predetermined coefficients in order to calculate velocities. Running the dynamic model is not usually computationally expensive and, therefore, it can generally be implemented on an existing device that already has access to the thruster command signals [48,51,52].

### 3.3. Acoustic Localisation Methods

Acoustic localisation is widely used in the marine environment, as acoustic waves propagate much further than electromagnetic waves in water [11]. In open water applications, acoustic localisation is a mature technology and it is the most important and reliable means of positioning and tracking available [53]. However, localisation in complex and confined environments brings a new set of challenges, which means that tried and tested hardware may not be suitable.

There are three main approaches to acoustic localisation which will be reviewed: Acoustic Beacons, Sonar SLAM (SSLAM), and Doppler Velocity Logs (DVLs).

All acoustic localisation relies on sound propagating through water between a reference object, or transducer, and the robot. Because the speed of sound in water is five orders of magnitude lower than RF propagation in air [11], all underwater acoustic systems will be affected by latency that is related to the time-of-flight: the time taken for a sound pulse to travel between the reference and the robot, and often back again. However, total latency remains acceptably low due to the relatively small environment sizes that were considered in this review. Assuming a nominal speed of sound of 1500 m/s and 20 m distance, this would give an inherent latency of approximately 26 ms for two way travel. Any time required for computation would be in addition to this.

A more problematic issue is that the ping rate (refresh rate) of the sensor is often directly related to the timeofflight. Typically, timeofflight systems require the current pulse to be sent and received and then any echoes must attenuate to a low level before the next pulse is sent. This means that the latency described above becomes the minimum possible loop time assuming no acoustic re-reflections. Using the example above, which would give a maximum sampling frequency of 38 Hz, which is low for a single range measurement.

In all acoustic localisation systems, there is a tradeoff between the range of the measurement and its resolution. This tradeoff is controlled by the system’s operating frequency. Low frequency waves propagate with less attenuation but their long wavelength reduces the resolution and precision of the derived measurements.

Another important factor to consider, especially in the present work, is that in an enclosed space, there is no option for the acoustic energy to dissipate into open water. This can cause increases in multipath, where an acoustic signal arrives at its target transducer via multiple different paths and the direct path does not always give the strongest response [11]. In addition, there is a possibility that there will be multiple rereflections (echoes) of the sound inside the enclosed water column that are caused by sound waves bouncing between the walls. Some acoustic energy will dissipate into the surrounding walls, with the amount depending on the acoustic impedance match between the water and surrounding wall materials. This raises the importance of the choice of operating frequency: higher frequency waves dissipate faster, which reduces the effect of multipath and rereflections. Conversely, lower frequency waves propagate better, giving higher range. The sampling rate can be increased by operating at multiple frequencies, but this has the negative effect of greater size, weight, cost, and complexity of the system.

These physical phenomena can have a drastic effect on the measurement accuracy of the system. A thorough investigation into the error sources present in acoustic localisation systems is provided by Taudien et al. [54].

#### 3.3.1. Acoustic Beacons

**Figure 3:**
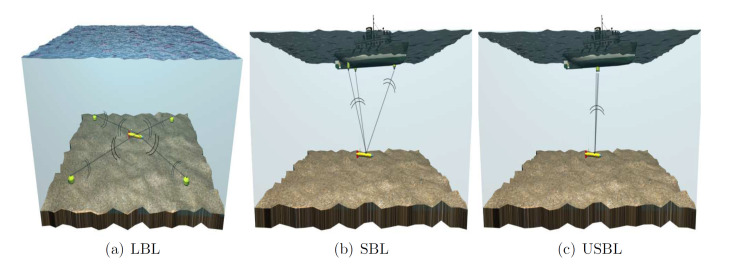
Classical underwater acoustic positioning systems for marine environments, reproduced from work presented by Alcocer et al. [56].

While GPS is not available underwater, localisation using fixed beacons, transponders, or transducers is the nearest equivalent and it has been the preferred method of underwater localisation in open water environments for many years [55]. Acoustic positioning using fixed beacons is a classical wave propagation and triangulation problem. The localisation system measures the timeofflight of the acoustic signal between the UUV and fixed transducer and can, therefore, estimate the distance between the transducer and the vehicle. The distance between the vehicle and several fixed beacons must be known in order to obtain a position fix in 3D space. In a marine context, traditional systems are often termed long base line (LBL), short base line (SBL) or ultra short baseline (USBL) systems (Figure 3); the difference being the distance between the fixed transducers.

One of the main attractions of localisation using fixed beacons is that the location is referenced to physical devices that are fixed in space. A drawback of this is that the accuracy of the localisation is reliant on accurately knowing the location of the fixed transducers in the global frame. Having just one transducer positioned incorrectly will have considerable impact on the overall accuracy of the system.

##### Suitable Hardware

The vast majority of commercially available systems are designed for open water applications and their size, accuracy, and cost are not suited to complex and confined spaces. Moreover, they are not specified for use in confined spaces and their accuracy may suffer considerably from issues, such as multipath. For instance, Teledyne’s Trackit USBL System [57] would usually be considered to be a short range system. However, its range of up to 1500 m is large relative to the environments considered in the present work (Section 2.2.2). The specified range accuracy of the Trackit USBL System is +/−0.3 m which is acceptable in most marine environments. However, in a nuclear fuel storage pool, for example, this level of accuracy would be too coarse for closed-loop control of a vehicle in close proximity to fuel rods.

Recently, the Ahoi-modem has been proposed as an inexpensive miniature approach to acoustic localisation and communication [58,59]. The cost is about 600 GBP per modem, of which the hydrophone’s share is 400 GBP leaving 200 GBP to the open-source PCB design. Its suitability for small-scale UUV localisation in a marina environment has recently been demonstrated in [60].

The only commercially available system known to the authors that is specified for operation in tanks or pools is the underwater GPS, produced by Water Linked [61]. The system is small enough to be fitted to the vehicles that are described in Section 2.4 and the cost is in the region of 4K GBP. The specified expected accuracy is 1% of the range between the locator and receivers, but there is limited information available regarding how this is affected by the dimensions and wall construction of the environment.

#### 3.3.2. Doppler Velocity Log—DVL

**Figure 4:**
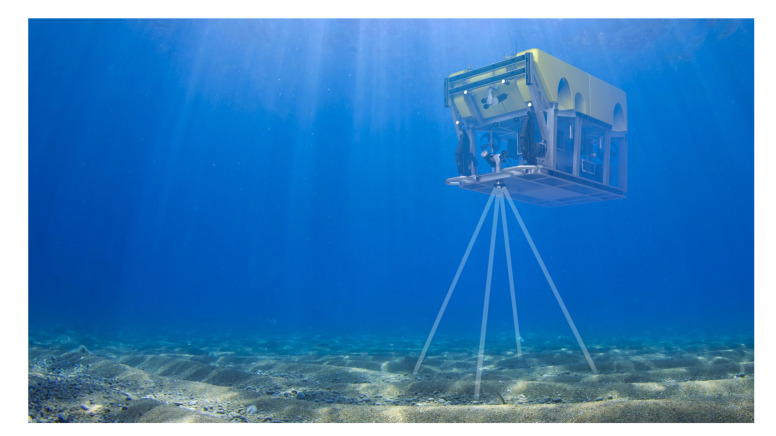
A schematic showing a Doppler Velocity Logs (DVL) being used to track the velocity of a UUV relative to the sea bed, reproduced from [64].

A DVL can be used in order to resolve the linear velocity of a vehicle relative to the ground. A standard DVL sends four fixed frequency acoustic beams downwards to the bottom of the water; each beam is offset to the the vertical by a known amount [62], see Figure 4. If the vehicle is in linear planar motion when compared to the ground, the frequency of the returning echo will be different from that of the transmitted signal. The difference, or Doppler shift, is proportional to the the speed of the vehicle over the ground [63]. If the vehicle velocity is provided by the DVL and heading is provided by an IMU or Attitude and Heading Reference System (AHRS), velocity in the global frame can be integrated over time to provide deadreckoning capabilities.

##### Suitable Hardware

DVLs are commercially available from a number of manufacturers such as Teledyne Marine or Nortek. Most units are designed for sub-sea operations and are large, expensive pieces of equipment e.g., [65]. However, in recent years, manufacturers have begun to produce smaller and lowercost units that would be suitable for use on smaller vehicles. The smallest DVL currently available is produced by Water Linked and has been demonstrated operating on the BlueROV2 in a water tank [66]. It has a specified range of 0.05–50 m, long term accuracy of +/−1 mm/s, and a ping rate of 4–26 Hz; which makes the unit suitable for confined space use.

#### 3.3.3. Sonar SLAM (SSLAM)

Sonar SLAM in an underwater environment is analogous to LiDAR based SLAM in a terrestrial environment [67]. Successive range scans are used inn order to estimate the vehicle’s position. Each scan is matched to a map which allows the vehicle’s position to be estimated relative to the map. The previous position is generally used as the starting estimate for the scan matching. Importantly, the map itself can be constructed by the algorithm in realtime while using the scan data.

Besides their high accuracy, the key advantage of a LiDAR sensor over sonar is that they gather each scan almost instantaneously [68]. Commercially available sonars that scan over a range wider that 180 degrees are generally of the mechanically scanned imaging sonar (MSIS) type. MSIS sensors emit and receive acoustic waves from a mechanical rotating head. The head rotates slowly and obtaining a complete scan often takes several seconds, in which time the vehicle has usually moved causing distortion. For this reason, and unlike commonly used LiDAR based SLAM algorithms, it is necessary to account for the vehicles motion between each individual range measurement. This requires the use of additional deadreckoning systems, such as those described in Section 3.1, Section 3.2 and Section 3.3.2.

SLAM systems localise relative to a map that is typically built by the algorithm and can be updated throughout the mission to cover newly explored areas. Despite the map updates, the transformation between the map frame and the global frame should not change significantly.

##### Suitable Hardware

To perform SSLAM in a confined underwater environment, a 360 degree MSIS is generally used [69]. MSIS sensors with ranges that are suited to confined space localisation are commercially available from a range of manufacturers such as Blue Robotics [70] or Tritech [71]. Although such units are commonly used in operations, they are often used in order to scan an environment and not for localisation, so their applicability for performing SSLAM in a confined space is largely unknown.

A major drawback of current SSLAM implementations is their reliance on MSIS sensors that have a slow scan rate, usually below 1 Hz. This could be potentially improved by the use of one or more wide-angle multi-beam sonars with higher scan frequencies, in the range of 10–50 Hz, e.g., [72].

The key benefit of SSLAM systems over fixed beacons is that no infrastructure needs to be deployed, calibrated or recovered. The SLAM localisation still references the fixed physical world around it and, so, it is not subject to the accumulation of errors. SSLAM could be a useful tool in confined space underwater localisation, although there is an absence of research where it is applied to smaller indoor confined spaces. A further consideration is that SSLAM systems rely on measurements from three or more sensors, which add to their cost and complexity.

#### 3.3.4. Summary on Acoustic Localisation

This section has introduced the three methods of acoustic localisation that are currently used on UUVs and has shown that they all have the potential for use in confined spaces, however their viability for specific scenarios is dependent on the environmental features that are discussed in Section 2. A full analysis of their suitability can be found in Section 4.

The technologies that have been discussed in this section are mature and they have been proven over years of use in successful subsea operations. In recent years, hardware that is typically used for long range localisation has been adapted for smaller vehicles and lower range use. The key issue at present is that there is virtually no literature that independently documents the use and accuracy of such systems when used in complex or confined environments. Therefore, the authors suggest that validating the applicability of these systems in the environments that are specified in Section 2.2.2 should be a priority in future research.

### 3.4. Visual Localisation

From the early days of mobile robotics, visual robot localisation has been one of the most widely used localisation technologies. Since then, the major challenge has been how to efficiently extract information rich data from the camera image stream. Early approaches aimed to extract lines and blobs within the image in order to reconstruct geometrical objects. These approaches still represent the basis for current state-of-the-art visual localisation concepts due to their mature robustness and advanced computational efficiency. However, more modern neural network-based concepts directly identify and track the object of interest without intermediate processing steps [73].

Generally, cameras are an appealing sensor for localisation tasks for various reasons. In comparison to other sensors, cameras are available at all price ranges, while providing information-rich data. Recent technological advances have boosted both the miniaturization and sensing quality of cameras, which makes their use by on-board embedded platforms attractive. Additionally, due to their passive nature, vision-based localisation systems do not suffer from the interference and multi-path effects that are common in acoustic and electromagnetic approaches. However, visual localisation requires there to be free line of sight between the sensing camera and the reference object of interest. In the context of submerged scenarios, light conditions, light absorption, and turbidity become key parameters when designing a vision-based localisation system. In addition, submerged environments often do not possess the feature richness of surface scenarios and there is also often a reduced texture quality rendering visual detection challenging. Despite these constraints, visual localisation constitutes a promising approach for confined underwater scenarios, where good visibility conditions are expected.

As with other localisation technologies, existing visual approaches can be split into a number of groups. First, concepts that define the robot position in a fixed absolute reference frame. This is usually the case when a-priori knowledge on landmark features is used. This group also includes approaches that require external infrastructure, such as multi-camera systems. The second group consists of concepts that describe the robot position relative to an initial reference frame, e.g., SLAM and Visual Inertial Odometry (VIO) approaches. These groups are not exclusive, as some techniques and algorithms cannot be uniquely assigned to one or the other group.

Concepts can be further sub-grouped into self-localisation techniques, where the robot itself determines its pose and external systems, where the mobile is tracked by an external hardware that makes the localisation estimate. External systems require a permanent communication link with the robot in order to provide the robot with continuous data e.g., to enable position feedback control.

#### 3.4.1. Augmented Reality Marker

Given feature-poor environments in underwater scenarios, the number of potential landmark-features that can be easily detected and tracked by a vision-based algorithm is limited. A common approach is to add artificial features to the underwater scenery. Augmented reality (AR) markers have become popular for object tracking tasks within many robotic disciplines, especially mobile robotics. While artificial features are usually impractical in open environments, such as oceans and lakes, this is not the case for confined scenarios, where the required effort for augmentation is limited by the bounded space of interest. An example is the early work of Carreras et al. [74] where a visually coded dot pattern was placed on a pool’s floor. This allows for the robot’s vision algorithm to accurately determine the robot’s position and orientation in an world reference frame bounded by the dimensions of the pool.

AR marker design can be grouped into two categories: spherical (reflective) markers and flat tag markers, which visually encode additional information such as an unique identification number. In general, AR marker approaches come with the drawback that the markers have to be placed prior to the robot deployment and within the robot’s work-space. However, they have been proven to significantly improve the localisation accuracy and robustness and minimize drift in position estimates.

Depending on the localisation method, the marker positions have to be known in a given reference frame, which can require additional effort for calibration measurements. However, this extra effort highly depends on the accessibility of the robot work-space. For example, marker placement in a controlled research tank likely comes with less effort than in a third-party marina.

The most simple markers are dots or spheres, whose color is considerably different from their surrounding. This allows for an easy detection from the camera image. Multiple markers are combined into known three-dimensional (3D) pattern-configurations, which are then extracted from the camera image, in order to distinguish the detected markers from one another. These marker patterns allow for the reconstruction of the 3D-pose between the marker set and the camera. An important point to note is that the combinatorial problem of marker correspondence grows exponentially with the number of visible markers, which makes the deployment challenging for large and complex scenarios. Today, reflective sphere markers are the most common approach. However, variants exist e.g., uniquely colored markers that can simplify the assignment process on the cost of processing colored images [75].

In contrast to the simple spherical reflective markers, flat tag marker can carry additional visually encoded information, such as a unique identification number. This reduces the effort to be invested in correspondence search and results in a more stable tracking performance. Popular examples include the ARTooKit [76] marker system, the various versions of the AprilTag family [77,78,79], and the ArUCo marker system [80], which have been originally developed for augmented reality applications in the field of computer vision.

In [81], the authors study various publicly available fiducial marker families (ARToolKit [76], AprilTags [77], ArUco [80]) in underwater environments. In their work, the authors focus on static configurations and considered various turbidity and lighting settings with respect to criteria, such as smallest detectable marker size, maximum camera-to-marker angle, maximum distance, and processing times. Overall, they conclude that the AprilTag library shows favorable performance given their criteria. However, the AprilTag family showed the slowest performance out of the evaluated libraries.

The performance evaluation of dynamic application of underwater AR marker tracking is reported in [82]. In their work, the authors study various AprilTag configuration and densities in a research tank setup. They discuss the suitability for agile robot maneuvering, also referred to as hydrobatics [83]. Given a sufficient a of markers available within the camera field of view, they report the localisation accuracy in the range of only a few centimeters.

**Figure 5:**
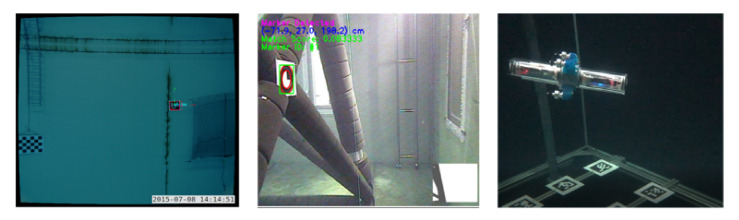
Example works using Augmented reality (AR) markers for underwater localisation, [85] (**left**), [86] (**center**), HippoCampus μAUV [82] (**right**).

There are many applications of fiducial marker systems. In [84], luminous ARToolkit markers are used to evaluate the performance of 3D camera pose estimation in an underwater scenario. The authors of [85] use a single wall-mounted AprilTag as a reference point for their underwater robot model identification algorithm. In [86], tag markers are mounted to a fixed infrastructure to allow for robot self-localisation relative to the structure for maintenance tasks (Figure 5). This work is adapted and extended in [87], where the authors sparsely place tags on the tank ground and use a SLAM algorithm in combination with a a downward looking camera to track ground objects in order to achieve accurate position tracking results.

A submerged autonomous manipulation task that is based on fiducial marker localisation is demonstrated in [88] with an Seabotix ROV. Within these experiments, the authors report a localisation accuracy with sub-centimeter accuracy for the robot’s translational degrees of freedom. A more dynamic navigation scenario is covered in [89] where the authors demonstrate the performance of their model predictive control scheme using a localisation scheme that based on fiducial pool-ground mounted markers and high-performance IMU.

Most of the reported literature use passive AR markers that are dependent on some kind of ambient illumination. Given the strong light absorption in water, passive markers naturally have reduced range in comparison to active illuminated markers. However, active markers require an individual energy source that comes with additional deployment and maintenance efforts for battery of external power supply. For this reason, only a few works are reported using illuminated markers, including the illuminated ArUco markers in [84] and the colored spherical markers in [75].

##### Suitable Hardware

Recent works have used a wide range of hardware configuration for AR vision-based self-localisation. A key criterion is whether image-processing and the subsequent localisation algorithms have to be implemented on-board the robot. This is naturally the case for scenarios where tetherless AUVs are deployed; an exception to this are external tracking systems as discussed in Section 3.4.2. In contrast, ROV platforms allow for the offloading of computationally heavy algorithms to remote computer workstations.

However, for on-board solutions, energy-efficient single board computer, such as the RaspberryPi family, Odroid boards, or Nvidia’s Jetson platform, are used in combination with commercial webcam-like cameras. Even these low-cost systems can achieve sub-centimeter to centimeter accuracy in localisation, despite the camera resolution often being reduced to 640×480 pixels, due to the limited computational power.

#### 3.4.2. External Tracking Systems

External visual localisation systems target two applications fields. First, ground truth reference tracking of underwater objects, e.g., to benchmark on-board self-localisation systems. Second, off-board robot localisation, which enables absolute position feedback control for robots that do not possess sufficient on-board localisation capabilities. In both cases, the suitability of external visual localisation systems is practically limited to confined scenarios that allow for the deployment of the camera infrastructure, such as tanks in research facilities. In this sense, these setups constitute the submerged dual of aerial and terrestrial robotic test-beds, such as the Flying Machine Arena at ETH Zurich [90,91] or Georgia Tech’s Robotarium [92].

These systems’ hardware typically consist of multiple high-speed tracking cameras [93,94], which are capable of tracking reflective marker at frequency up to 350 Hz and with millimeter accuracy. Therefore, the markers have to be mounted in a known pattern on-board the robot. Given this known 3D marker configuration, the algorithms search for marker correspondence in order to minimize the re-projection error between the detected marker and predicted marker positions. Based on this measurement, the algorithm estimates the relative pose between marker setup and camera.

In the context of reference tracking, this can also be done in an online or offline manner. An online, low-cost approach to an underwater multi-camera systems for reference tracking is proposed in [95]. Here, the authors track a micro underwater robot that is equipped with AprilTags in a confined research tank (4×2×1.5 m${}^{3}$). They use multiple webcam sensor nodes and decentralized RaspberryPi single board computer. In [19], the authors use an external webcam and coloured LEDs to track an ROV to allow for reconstruction of sonar images of objects on the bottom of a test tank. An offline approach is presented in [96], where action sports cameras are used to track and analyze the motion of a human swimmer.

Similar to the corresponding test-beds in aerial robotics, these systems can also be used to close the loop for robot position feedback control by providing the underwater robot with information on its absolute position. Examples include the *SCUUL* test-bed at the Neutral Buoyancy Research Facility at University of Maryland [97] (15 m diameter, 8 m depth). However, these off-board tracking setups require a continuous data link to the underwater vehicle, which is subject to very limited bandwidth and high latencies and, thus, constitute a bottleneck for agile underwater robot control. A reduced tracking setup is reported in [98], where the authors use a single birds-eye camera to provide a swarm of robotic fish with their position information within a small-size swimming pool.

**Figure 6:**
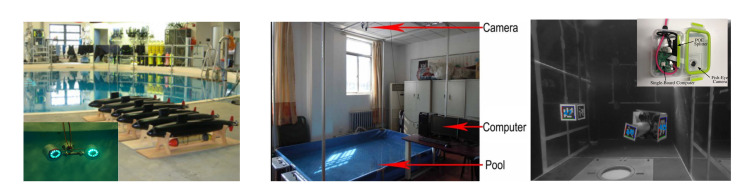
Example works external camera systems for underwater localisation, SCUUL multi-camera testbed [97] (**left**), Single-topdown camera [98] (**center**), low-cost multi-camera system [95] (**right**).

Figure 6 depicts a selection of existing approaches.

##### Suitable Hardware

External tracking systems usually rely on multiple commercial high-speed tracking cameras [93,94]. The number of deployed cameras directly correlates with the required workspace volume and the desired accuracy, since a higher number of cameras provide a more accurate, robust, and smooth tracking performance. These cameras usually feature blue LED spotlights in order to extend the maximum detection range between marker and camera. The detection range is correlated with the deployed camera equipment. For 19 mm markers, detection ranges of 14–27 m have been reported, depending on the camera module [93]. The price range of these cameras lies around GBP 4k per camera. However, low-cost approaches are available, such as the open-source RaspberryPi- and ROS-based multi-camera reference tracking system proposed in [95] with a cost in the range of GBP 50 per camera node. Moreover, an offline-tracking methodthat is based on sport actions cameras is presented in [96].

#### 3.4.3. Vision-Based SLAM

Vision-based SLAM algorithms are a powerful tool for self-localisation in unknown environments. Traditionally, most works focus on applications in terrestrial and aerial domains. However, research on SLAM methods for submerged scenarios has gained considerable interest in recent years [99,100,101,102]. While existing methods can usually be transferred to underwater applications, domain specific properties, such as feature-poorness and ambiguity, have to be considered in the development process.

Various projects have targeted the application case of submerged ship-hull inspection [99,100]. In [99] a visual pose-graph SLAM approach is used, which the authors benchmark against dead reckoning navigation.

In comparison to sonar SLAM, see Section 3.3.3, vision-based algorithms, given sufficient underwater visibility, enable higher resolutions and provide more accurate position information. However, this comes at the cost of a reduced sensing range in comparison to acoustic systems. Therefore, various works [100,102] combine visual and sonar SLAM in order to benefit from their technology-specific advantages. While [100] studies a ship-hull inspection scenario, the recent work presented in [102] studies visual-acoustic SLAM for underwater cave mapping.

SLAM tracking performance strongly relies on the quality of the detected features. Thus, AR markers, as discussed in Section 3.4.1, can be deployed to improve tracking accuracy and robustness. An AprilTag-based SLAM approach is presented in [101]. This SLAM-AR-marker combination is appealing, since it does not require pre-mission calibration of the deployed AR-marker. Moreover, this allows for AR-markers to be used with SLAM algorithms as a reference benchmark for other SLAM algorithms when the AR marker are placed along the trajectory.

All of the previous introduced methods rely on submerged visual features. An interesting multi-domain approach is presented in the recent work conducted by Suresh et al. [103]. In their work, the authors propose a SLAM concept for confined environments. The underwater robot runs a SLAM algorithm that relies on terrestrial visual features. The authors propose a stereo-camera SLAM algorithm that allows an underwater robot with top-mounted cameras to track its position through the water surface against the ceiling of the pool surrounding building.

##### Suitable Hardware

The hardware requirements of visual underwater SLAM methods mainly differ with respect to their camera sensing concept—monocular vs. stereo—and to what extend they rely on additional sensors. Rovco have developed a commercially available system, the SubSLAM X2 [104], which uses stereo-cameras as the basis of the vision system. This system has a small form factor; 0.4 m × 0.275 m × 0.14 m, and it is able to be mounted on smaller UUVs, such as the BlueRoV2. A significant challenge to overcome with VSLAM systems is the computational requirements and being able to do this on-board the UUV for autonomous missions. If the SLAM algorithm is to be used on-board a tethered small-scale ROV, it may be necessary to offload the extensive computations to an external computing unit with independent energy supply.

#### 3.4.4. Summary on Visual Localisation

This section has introduced the three fundamental methods of visual localisation and has shown that all of them can be potentially used in confined spaces. However, their individual viability for specific scenarios is strongly dependent on the environmental features discussed in Section 2. A full analysis of their suitability is presented in Section 4.

For non-marine robots, vision-based localisation is often seen as the most important source for information on the robot localisation. This is particularly the case for localisation in terrestrial and aerial robots. The concepts that are discussed in this section are well studied and widely deployed throughout various domains.

However, transferring and adapting visual localisation methods from non-marine applications to the specific challenges of confined submerged environments, as discussed in Section 2.2.2, is still a hard step and there is significant research still to be undertaken.

### 3.5. Electromagnetic Localisation

Electromagnetic (EM) localisation constitutes a niche method for short-range distancing, as required in confined environments and is not widely covered in the available literature surveys on underwater localisation.

The concept of underwater robot localisation based on the attenuation of electromagnetic (EM) carrier signal waves for short range localisation has been explored in a recent series of publications [105,106,107,108,109,110,111]. EM-localisation exploits the strong attenuation of EM-carrier signals in water, which is otherwise usually undesirable e.g., for communication. Due to its limited range, it primarily targets strictly confined scenarios with length scales <5 m, such as docking [112] or maneuvering in small tanks [110]. The carrier signal attenuation is described by an a-priori identified range sensor model (RSM), which allows for the computation of the distance between an emitting anchor node and a receiver on-board the robot, based on the received signal strength (RSS).

**Figure 7:**
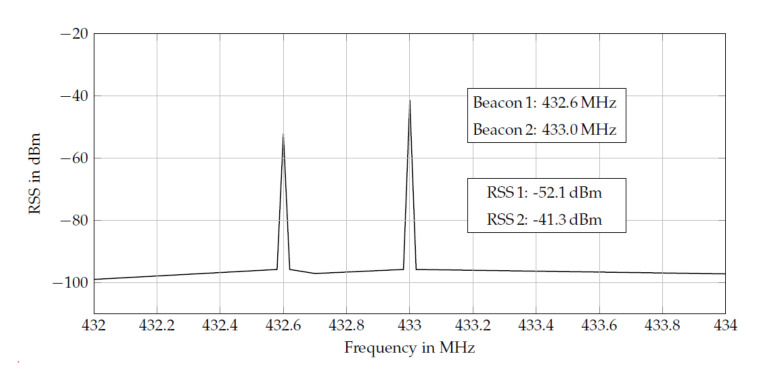
Signals from two beacons emitting at 432.6 MHz and 433.0 MHz. The receiver identifies the emitting beacons based on the frequency and determines the range to the beacons using the received signal strengths (RSS) values.

EM-carrier signals are emitted by spatially distributed anchor nodes at known positions. Each anchor node emits its signals at an unique frequency channel. A mobile node (e.g., the robot) captures the overlayed power spectrum density. A fast Fourier transformation is then applied in order to extract the individual anchor’s received signal strengths (RSS) from the power spectrum. Exploiting the channel allocation, the extracted RSS values can be assigned to the individual anchor node based on their unique frequencies (Figure 7). This allows for the processing of all incoming RSS data within a single step, rather than sequential node-wise processing. Various EM-frequency bands are possible and longer wavelengths allow for extended detection ranges. However, known approaches [107,108,109,110] favor the commercial 433 MHz frequency band since suitable transmission and receiving devices are available off-the-shelf.

In the original work [105], an underwater RSM is introduced exploiting Frii’s transmission formula and the underlying Maxwell equation. When modelling the signal, attenuation, conductivity, permeability, and permittivity parameters of the transmission medium have to be taken into account. In addition, the emitted and received signal strength is strongly affected by the antenna design.

However, Park et al. show, in [105], that these individual parameters can be summarized by introducing an environmental parameter Γ and Λ. This results in the simplified RSM for the planar case

(1) $$\begin{array}{c} {\mathrm{RSS}}_{i}=-20\;\log_{10}\left(R_{i}\right)-20\;R_{i}\;\alpha \;\log_{10}\left(e\right)+\Gamma \left[\mathrm{dBm}\right], \end{array}$$

where ${\mathrm{RSS}}_{i}$ denotes the received strength of a carrier signal emitted by anchor node *i*, received by the mobile robot anchor at distance $R_{i}$, alpha is the propagation constant and Γ summarizes all unmodelled constant effects. Given this model in combination with an external high-fidelity spectrum analyzer, the authors of [106] report sub-centimeter accuracy at 1 KHz update rate for position tracking experiment in a workspace of approximately 2×1.5m.

So far, the RSM, as defined in Equation (1), is limited to model the carrier signal attenuation within the plane defined by the anchors. In [107], the RSM is extended in order to capture varying relative orientations between the anchor’s transceiver antenna and the robot’s receiver antenna. Subsequently, the concept is extended to 3D by stacking multiple anchor nodes and providing the robot with depth sensor data, as studied in [108]. These early works used an external full-fledged spectrum analyzer (NI 5660 vector spectrum analyzer) to compute the power density spectrum in real-time. The rack-size spectrum analyzer makes this setup unsuitable for small size UUV deployment for size and cost reasons. Therefore, when using an off-board spectrum analyzer, a tether connection to the robot’s antenna is required, which limits the deployment to tethered ROVs.

### 3.6. Suitable Hardware

**Figure 8:**
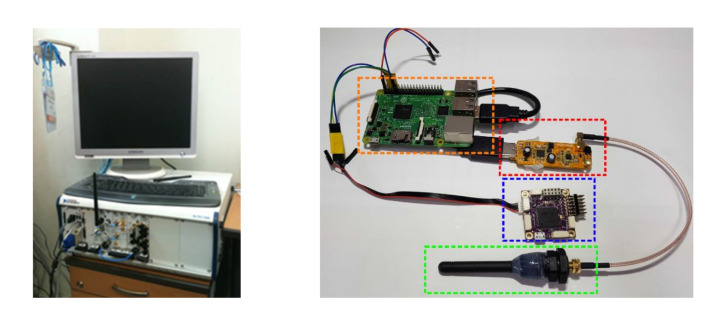
Hardware setups for EM-based localisation; using an external vector spectrum analyzer [105,106] (**left**) and the miniaturized DVB-T USB-dongle SDR-based concept with single board computer by [109,110] (**right**).

This size and cost challenge is addressed in [109], where the authors present a small scale and low-cost approach to EM-based localisation targeting deployment in micro UUVs. They replace the large and expensive spectrum analyzer by a digital video broadcast (DVB-T) USB dongle, which is capable of software defined radio (SDR) and a RaspberryPi single board computer (SBC), see Figure 8. Using the light-weight setup, the authors demonstrate centimeter localisation accuracy at 5–15 Hz update rate, depending on the SBC’s computing capabilities. The suitability of this setup for position feedback control on-board a micro AUV is demonstrated and critically discussed in [110] for two confined scenarios.

## 4. Discussion

Localisation technologies that are based on four different physics principles have been identified and reviewed in this article. Eleven environmental characteristics were outlined in Section 2.2.2. Table 5 shows the relationship between some of these characteristics and the underlying principles and also summarises some common comparison metrics. Table 6 shows the suitability of different technologies for localisation within the environments that are discussed in Section 2.2.1, ranked as either low, medium, or high.

A key consideration in the selection of a localisation technology is the requirement for additional infrastructure. In complex and confined environments, additional infrastructure may be very difficult to deploy, especially if sub-surface beacons are required to be placed around the perimeter of the environment. Fixing and accurately locating beacons is a time consuming task and, if beacon locations are not correctly set, this will have a significant negative impact on the accuracy of the localisation estimate. Fixing beacons to the environment can also be challenging in certain application environments, such as nuclear, due to access restrictions and the increased risk of contamination.

An alternative solution, where surface access is available, may be to mount these beacons on autonomous surface vehicles (ASVs), much in the same way as ocean-going localisation systems are often referenced back to a deployment boat. The same challenges with accuracy of the beacon placement are present, as the ASV will not be fixed to the environment.

**Table 5:** Technology Comparison.

Criteria	Acoustic			Dynamics		Electromagnetic	Vision		
	Acoustic Beacons [61]	DVL [66]	Sonar SLAM [68]	Inertial MEMS IMU [45]	Dynamic Models	EM-Signals [108,109]	AR Tags [82]	Ext. Tracking [93,95,97]	VSLAM
Local. Type	Absolute	Relative	Absolute	Relative	Relative	Absolute	Absolute	Absolute	Relative
Stand Alone	Y	Y	N	N	N	Y	Y	Y	N
Range	100 m	Infinite	75 m	Infinite	Infinite	2 m	5–10 m	10–30 m	5 m
Reflections	Severe	Mild	Mild	Unaffected	Unaffected	Mild	Low	Low	Low
Add. Infra.	Y	N	N	N	N	Y	Y	Y	N
Infra. Size	27 × 24.6 × 12.4 cm	N/A	N/A	N/A	N/A	20 × 10 × 10 cm	10 × 10 × 1 cm	25 × 15 cm	N/A
LoS	Important	N/A	N/A	N/A	N/A	Important	Important	Important	N/A
Turbidity	Unaffected	Unaffected	Unaffected	Unaffected	Unaffected	N/A	Important	Important	Important
Amb. Illum.	Unaffected	Unaffected	Unaffected	Unaffected	Unaffected	N/A	Important	Important	Important
Salient Features	N/A	Mild	Medium	N/A	N/A	N/A	Important	N/A	Important
Cost	≈5K GBP	≈6K GBP	≈5K GBP	≈3K GBP	N/A	100–12k GBP	≈100 GBP	500–20k GBP	500 GBP
On-board Size	20 × 41 mm	66 × 25 mm	56 × 79 mm	5 × 5 × 1 cm	N/A	10 × 6 × 5 cm	10 × 6 × 3 cm	N/A	10 × 5 × 5 cm
Power	5 W	4 W	4 W	3 W	N/A	5 W	5 W	5–20 W	10 W
Update Rate	2–4 Hz	4–26 Hz	5–20 s	4 kHz	Variable	10 Hz/1 kHz	10–30 Hz	100–300 Hz	10 Hz
Accuracy	≈1% of range	±0.1 cm/s	±1.9 m	±100 km/h	Variable	±1–5 cm	±1–5 cm	±1 cm	variable

**Table 6:** Suitability of localisation Technologies for Different Applications.

Area	Acoustic Beacons	DVL	Sonar SLAM	Inertial	Dynamic Models	EM-Signals	AR Tags	External Tracking	VSLAM
Modern Nuclear Storage Pond	Low	Med	Med	Med	Med	Low	Med	Med	Med
Legacy Nuclear Storage Pond	Low	Med	Med	Med	Med	Low	Low	Med	Med
Legacy Nuclear Storage Silo	Low	Low	Low	Med	Med	Low	Low	Low	Low
Nuclear Reactor Pressure Vessel	Low	Low	Low	Med	Med	Low	Low	Low	Low
Offshore Asset Decommissioning	Low	Low	Med	Med	Med	Low	Low	Low	Med
Ship Hulls	Med	Low	Med	Med	Med	Low	Med	Low	High
Liquid Storage Tanks Marinas, Harbours and Boatyards	Low	Low	Low	Med	Med	Low	Low	Med	Med
	High	Med	Med	Med	Med	Low	Med	Med	Med
Tunnels, Sewers and Flooded Mines	Low	Med	Med	Med	Med	Low	Low	Low	Med

### 4.1. Acoustic-Based Systems

Acoustic systems are the most widely used in ocean-based localisation and, as such, they have the largest range. Unfortunately, when they operate in confined environments with obstacles, they suffer significant interference from multi-path propagation. However, they are not affected by visual spectrum interference. They can provide either an absolute or relative position estimate.

#### 4.1.1. Acoustic Beacons

Table 6 shows that this technology is only suitable for use in marinas, harbours, and boatyards. The two primary challenges with the use of this technology are the inability to place external infrastructure and the severe multi-path which is generated in cluttered and confined areas.

Line of sight is an important factor in cluttered environments. Loss of line of sight may be overcome by the use of mobile beacons, however this adds an additional layer of complexity to the system. Alternatively, SLAM-based systems could be used when line of sight is lost.

The scarcity of suitable commercially available systems and independent testing of these systems in different complex and confined environments is also a limitation. A custom built system with an operating frequency that is tuned to match the environment could potentially improve performance; however, this will only mitigate the physical constraints so much.

Whilst acoustic beacons are the default localisation technology for ocean-based systems, they are generally unsuitable for most confined environments.

#### 4.1.2. DVL

The use of DVLs is commonplace in marine applications [51,68,69,113,114]. They are typically used alongside a means of absolute localisation to join up regions between location fixes or to aid dynamic positioning or station keeping of aquatic vehicles. This would also be the case for confined space localisation.

As a minimum, a DVL requires a method of obtaining the heading of the vehicle for localisation to be achieved in a global frame. The introduction to the market of new hardware that is specifically designed for use on smaller vehicles means that the use of DVLs in confined space localisation may now become much more prevalent.

Because these products are relatively new, they have not had the many years of testing and real world validation that larger units have had in sub-sea operation [113], so more detailed conclusions regarding their suitability cannot be drawn yet.

#### 4.1.3. Sonar SLAM

Ribas and collaborators have repeatedly demonstrated that SSLAM can be used to localise a vehicle in an underwater environment [67,68,69,115,116,117]. Testing in an abandoned marina, Mallios et al. found that their posebased SLAM method has a mean accuracy of 1.9 m [68]. In these experiments, the SSLAM system was aided by dead reckoning from an IMU and a DVL, which is a typical arrangement.

This level of error is relatively large when compared to many of the confined environments that are the focus of this review. However, the marina environment used in the experiments was large, with range measurements frequently exceeding 50 m. A reasonable assumption can be made that accuracy could be considered as a percentage of the range measurements, which would give an accuracy of 4% based on an estimated average 50 m range measurement.

The key benefit of a SSLAM system over fixed acoustic beacons is that no infrastructure needs to be deployed, calibrated, or recovered and the SLAM localisation still references the fixed physical world around it, so is not subject to accumulation of errors.

SSLAM could be useful tool in confined space underwater localisation, although there is an absence of research where it is applied in smaller indoor confined spaces. An important note to make, is that typical SSLAM systems rely on measurements from three or more sensors, which add to their cost and complexity and may not fit on some of the smaller UUVs identified in Section 2.4.

### 4.2. Dynamic Localisation Systems

Dynamic localisation systems (inertial sensing or system models) can only be used in conjunction with one of the other technologies. As they only uses proprioceptive sensors, they are robust to many of the environmental constraints; however, because they only provide a relative position estimate while taking prior information into account, the errors and uncertainties can grow rapidly, unless corrected by an absolute localisation system.

#### 4.2.1. Inertial Systems

Inertial navigation is used widely in subsea environments and often forms an integral part of the localisation package [63]. In [118] Rogne et al. evaluate the use of MEMS inertial sensors on a marine vessel that has closedloop control of its position and heading (dynamic positioning). They find that MEMS IMUs are a valuable addition, either as a replacement or to compliment existing systems such as the DVL. Because inertial navigation is largely environment agnostic, it is equally suited to localisation in confined underwater spaces. However, in a confined underwater environment, the required positioning accuracy is generally much higher than in an open-ocean environment; therefore, selecting an IMU with the lowest possible in-run bias instability is recommended.

Because of the high drift of suitable IMUs, the use of an unaided inertial navigation system is not generally recommended in confined environments. Instead, the inertial system should be used to compliment other technologies. For example, in [51,52], the dead reckoning capabilities of inertial navigation systems are improved by fusion with a dynamic system model and in [68,119,120,121] the performance of absolute positioning systems are improved by the incorporation of an IMU.

#### 4.2.2. Dynamic Models

The most common use of dynamic models in aquatic vehicles is to aid in understanding and design of control systems that automatically hold aquatic vehicle positions and velocities, e.g., [122,123,124,125]. However, there are also many examples where aquatic vehicle localisation is achieved or aided by a dynamic model [50,51,52,126,127]

Localisation from a dynamic model is particularly attractive in confined spaces, because water currents are not always present and circulation pumps can often be temporarily turned off. The fact that, in most cases, no added hardware is required is also a very positive point due to tight space, weight, and cost restrictions on small vehicles. However, the use of an unaided dynamic model for localisation is not recommended, due the dynamic model’s high susceptibility to disturbances and the accumulation of errors. Rather, a dynamic model is suited to aiding existing systems in order to improve precision or to deadreckon between position fixes.

### 4.3. EM-Signals

EM-signal systems only operate over a short range, but have high accuracy. They require additional infrastructure to be deployed, but they are not affected by visual or acoustic spectrum interference.

**Figure 9:**
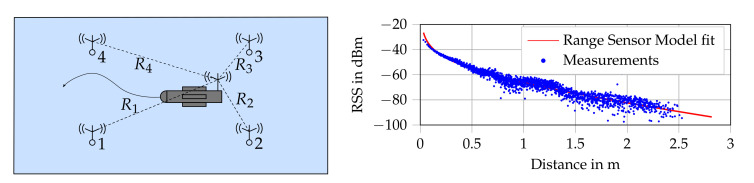
EM-localisation concept with four emitting anchor nodes and a receiver onboard an UUV (**left**) and range sensor model fit from calibration measurements (**right**).

Although promising results have been presented using EM-localisation in confined scenarios, EM-based localisation still face various challenges. Depending on the confined environment, existing methods are subject to carrier signal reflection, e.g., caused by tank walls. These reflections distort the monotonically decreasing signal strength attenuation (Figure 9) and, thus, reduce the localisation accuracy.

Another challenge is the sensitivity towards antenna orientation mismatches between the transmitter and receiver, as discussed in [107,110]. Kwak et al. [107] aim to model this drop of RSS by high order sine and cosine terms. However, small scale UUVs naturally come with more agile dynamics than large ROVs and demand for a robust dynamic compensation of the relative antenna orientation. Further work is required in order to study and extend existing EM-localisation methods towards micro underwater robots.

Due to the absence of physical antennas with isotropic attenuation pattern, dipole antennas with omni-directional patterns are used. So far, this renders consistent full 3D EM-localisation coverage challenging. However, depending on the specific scenario, stacked anchor arrays, as proposed in [108], can be used.

For the environments that are covered in Table 6, EM-Signal localisation is not suitable for any of them as the primary method of localisation. This approach could, however be used in a more targeted manner in scenarios where high positioning accuracy for high precision robot maneuvering within an a-priori known area, for example docking.

### 4.4. Vision-Based Systems

Given confined scenarios, vision systems have the best balance of accuracy to range of all of the technologies considered. However, they are highly susceptible to environmental conditions, such as turbidity, illumination levels, optical reflections, and the availability of salient features. Depending on the method used, vision-based systems can provide either an absolute or relative position estimate.

#### 4.4.1. AR Tags

Augmented reality marker systems constitute an attractive choice to achieve highly accurate and robust localisation in scenarios where there is sufficient visibility and limited turbidity, but that does not possess sufficient visual features. Their major drawback is the required effort of pre-mission deployment.

However, depending on the environment, those efforts have to be invested only once if the marker infrastructure can remain within the confined volume, e.g., if tag markers are painted on tank walls. This is usually the case for dedicated facilities, such as research tanks, but it may be the case for others.

A trade-off exists between the required detection range of the markers and the marker dimension. The detection range and, thus, the work-space covered by the localisation systems, can be additionally increased by using illuminated markers and better camera sensors. Successful detection ranges have been reported up to 10 m between camera and marker in good operating conditions.

If localisation methods are used that require exact knowledge of the marker position, the effort for this calibration process can be significant. In this case VSLAM methods, as discussed in Section 3.4.3, can be a convenient alternative if high accuracy is less not the top priority.

For the environments that are presented in Table 6, AR tags could be used in modern nuclear storage facilities, ship hull inspections or marinas, harbours, and boatyards. For all of these environments, the AR tags could be installed prior to inspections. For example, the tags could be placed on the sides of nuclear storage containers and coupled with consignment tracking tags. For the other unsuitable environments, the inability to prior locate the AR tags means that they are not feasible to use.

#### 4.4.2. External Tracking

External vision-based tracking systems are common for sophisticated robotic test-beds, where they provide accurate robot position tracking at high-rates. External tracking systems naturally require the deployment of highly calibrated infrastructure. Therefore, their general suitability is limited to confined scenarios that allow long-term deployment of additional equipment, the same problem as with acoustic beacons.

However, there is ongoing research into their use with lower-quality systems requiring less calibration mounted on floating platforms. This could prove to be highly effective where there is surface access, such as nuclear storage ponds.

#### 4.4.3. VSLAM

Visual SLAM algorithms are an attractive and common choice for navigation in unknown environments, as they do not rely on additional infrastructure. However, their performance can be improved when additional features, such as AR markers, are deployed. An attractive advantage of VSLAM systems is the map generation of the potentially unknown environment, which can also be used for planning tasks.

As with traditional non-aquatic VSLAM systems, they are reliant on salient features in the environment to be able to be detected. When these are not available, the system will not work properly and a back-up localisation method may be required.

In direct comparison to acoustic approaches, VSLAM is usually more accurate while facing a shorter sensing range. Therefore, both sensing concepts are often combined. Furthermore, VSLAM systems usually fuse IMU and DVL data in order to improve their tracking performance. This results in a high cost of SLAM systems, since they combine various expensive sensing components.

Overall, VSLAM could be used in a range of environments, if the water quality is high.

## 5. Conclusions

This article has presented a review of the state-of-the-art in underwater localisation technologies from the perspective of being used in complex and confined environments. Significant research has been undertaken into aquatic localisation for use in the open-ocean; however, these technologies are very often not transferable to confined spaces.

The default localisation technologies in the open-ocean are acoustic based; however, these are often not suitable for confined environments due to the severe multi-path which occurs. Dynamics-based systems can only provide relative position estimates, but they can be universally used and fused with other systems, whilst EM-signal systems can only be used over very short distances. Vision-based systems offer the best solutions, either external tracking or VSLAM, however they quickly fail if the water quality is poor.

Rather than a single solution, it is likely that multi-modal localisation systems will be required to robustly operate in complex and confined environments [128].

Nine complex and confined environments were identified across a range of industries. For modern and legacy nuclear storage ponds and offshore asset decommissioning, external vision tracking systems using cameras that are mounted on ASVs may be the most suitable technology. This could be coupled with VSLAM, DVL, or SSLAM. In modern nuclear storage ponds, water clarity is kept very high; however, clarity can be variable in legacy nuclear storage ponds, so having multi-modal localisation (vision and acoustics for example) may be necessary.

There are no suitable absolute localisation technologies available for legacy nuclear storage silos and nuclear reactor pressure vessels. This is highlighted by the challenges surrounding the inspection of the Fukushima Daiichi Nuclear Power Plant in Japan, where ROVs have been deployed, but they have been unable to be localized and, therefore, the data that have been returned cannot be geo-spatially referenced, which is required for the decommissioning process. Novel localisation technologies will need to be specifically developed for these environments.

Liquid storage tanks are another challenging environment due to the restricted access and inability to install external infrastructure. VSLAM may be the most suitable technology.

Ship hulls, marinas, harbours, boatyards, tunnels, sewers, and flooded mines are all on the larger in scale, which provides more options. Ship hull, marinas, harbours, and boatyards are on the threshold of ocean/confined and it may be easier to adapt some of the ocean-based technologies, such as acoustic systems. Tunnels, sewers, and flooded mine inspections happen over longer distances (although the width of the environment may be constrained), so DVLs could be used. VSLAM could also be used if the water quality was good enough.

Overall, significant research and development is still required to accurately and reliably localize UUVs in complex and confined environments. There is no single technology that could be applied to all environments and new disruptive technologies are required in the case of legacy nuclear storage silos and nuclear reactor pressure vessels.

## Figures and Tables

**Table 1:** Environmental Characteristics of Confined and Hazardous Application Areas.

Area	Scale (m)	Obstacles	Structure	Obstacle Type	Access	Additional Infrastr.	LoS	Turbidity	Ambient Illumin. Levels	Salient Features	Variance of Env.
Modern Nuclear Storage Ponds	50 × 100 × 10	Y	Structured	Both	Surface	Maybe	Surface	V. Low	Good	Med	V. Low
Legacy Nuclear Storage Ponds	50 × 100 × 10	Y	Unstructured	Both	Surface	N	Surface	Variable	Good	Med	Med
Legacy Nuclear Storage Silos	5 × 10	Y	Unstructured	Static	Restricted	Surface	Maybe	Variable	None	Low	Med
Nuclear Reactor Pressure Vessels	5 × 10	Y	Unstructured	Static	Eith	N	None	V. High	None	Low	V. Low
Offshore Asset Decommissioning	5–50	Y	Unstructured	Static	Either	N	Maybe	Variable	Low	Med	Low
Ship Hulls	10 × 50 × 2	Y	Structured	Static	Surface	Maybe	Both	Variable	Med	Med	Low
Liquid Storage Tanks Marinas Harbours and Boatyards	20 × 30	N	Structured	Static	Restricted	N	Both	Low	None	Low	V. Low
	100 × 100 × 10	Y	Both	Both	Surface	Maybe	Both	Variable	Med	Med	V. High
Tunnels, Sewers and Flooded Mines	3 × 3 × 100	Y	Both	Static	Either	N	None	Variable	None	Med	High

**Table 2:** Current Mission Types and Levels of Autonomy (LoA) for Different Applications.

Area	Inspection	Maintenance/ Repair	LoA0	LoA1	LoA2	LoA3	LoA4	LoA5
Modern Nuclear Storage Pond	N	N						
Legacy Nuclear Storage Pond	Y	Y	X	X				
Legacy Nuclear Storage Silo	Y	N	X					
Nuclear Reactor Pressure Vessel	Y	N	X	X				
Offshore Asset Decommissioning	Y	Y	X	X				
Ship Hulls	Y	N	X	X				
Liquid Storage Tanks Marinas, Harbours and Boatyards	Y	Y	X	X				
	Y	N	X	X				
Tunnels, Sewers and Flooded Mines	Y	N	X	X				

**Table 3:** Predicted Mission Types and Levels of Autonomy for Different Applications.

Area	Inspection	Maintenance/ Repair	LoA0	LoA1	LoA2	LoA3	LoA4	LoA5
Modern Nuclear Storage Pond	Y	Y	X	X	X	X	X	X
Legacy Nuclear Storage Pond	Y	Y	X	X	X	X	X	
Legacy Nuclear Storage Silo	Y	Y	X	X				
Nuclear Reactor Pressure Vessel	Y	Y	X	X	X	X		
Offshore Asset Decommissioning	Y	Y	X	X	X	X		
Ship Hulls	Y	Y	X	X	X	X	X	X
Liquid Storage Tanks Marinas, Harbours and Boatyards	Y	Y	X	X	X	X	X	X
	Y	Y	X	X	X	X		
Tunnels, Sewers and Flooded Mines	Y	Y	X	X	X	X	X	X

**Table 4:** Characterisitcs of UUVs.

UUV	Dims. l × w × h (m)	Depth Rating (m)	Tethered	Payload (kg)	Battery Life (hrs)	Commercial/ Research
DTG3	0.28 × 0.33 × 0.26	200	Y	Unknown	8	C
VideoRay Pro 4	0.38 × 0.29 × 0.22	305	Y	Unknown	N/A	C
AC-ROV 100	0.2 × 0.15 × 0.15	100	Y	0.2	N/A	C
BlueROV2	0.46 × 0.34 × 0.25	100	Y	1	2–4	C
UX-1	0.6 dia	500	Y	Unknown	5	R
AVEXIS	0.15 dia × 0.3	10	Y	1	N/A	R
HippoCampus	0.15 dia × 0.4	10	N	1	1	R

## Abbreviations

The following abbreviations are used in this manuscript:

AHRS	Attitude and Heading Reference System
AR	Augmented Reality
ASV	Autonomous Surface Vehicle
AUV	Autonomous Underwater Vehicle
DOF	Degree of Freedom
DVB-T	Digital Video Broadcast
DVL	Doppler Velocity Log
EM	Electro-magnetic
GPS	Global Positioning System
IMR	Inspection, Maintenance and Repair
IMU	Inertial Measurement Unit
LBL	Long Base Line
LOA	Level of Autonomy
LoS	Line-of-Sight
MEMS	Micro-Electro-Mechanical Systems
MSIS	Mechanically Scanned Imaging Sonar
ROV	Remotely Operated Vehicle
RSM	Range Sensor Model
RSS	Received Signal Strength
SBL	Short Base Line
SDR	Software Defined Radio
SLAM	Simultaneous localisation and Mapping
SSLAM	Sonar-based Simultaneous localisation and Mapping
USBL	Ultra Short Base Line
UUV	Unmanned Underwater Vehicle
VIO	Visual Inertial Odometry
VSLAM	Vision-based Simultaneous localisation and Mapping
